# Systemic Characterization of Novel Immune Cell Phenotypes in Recurrent Pregnancy Loss

**DOI:** 10.3389/fimmu.2021.657552

**Published:** 2021-05-28

**Authors:** Hong Liu, Xin-Xiu Lin, Xiao-Bo Huang, Dong-Hui Huang, Su Song, Yang-Jiao Chen, Jing Tang, Ding Tao, Zhi-Nan Yin, Gil Mor, Ai-Hua Liao

**Affiliations:** ^1^ Institute of Reproductive Health, Center for Reproductive Medicine, Tongji Medical College, Huazhong University of Science and Technology, Wuhan, China; ^2^ Center for Reproductive Medicine, Tongji Medical College, Huazhong University of Science and Technology, Wuhan, China; ^3^ Department of Gynecology, Maternal and Child Health Hospital of Hubei Province, Wuhan, China; ^4^ School of Data Science, The Chinese University of Hong Kong, Shenzhen, China; ^5^ Zhuhai Precision Medical Center, Zhuhai People's Hospital, Jinan University, Zhuhai, China; ^6^ The Biomedical Translational Research Institute, Faculty of Medical Science, Jinan University, Guangzhou, China; ^7^ C.S. Mott Center for Human Growth and Development, School of Medicine, Wayne State University, Detroit, MI, United States

**Keywords:** recurrent pregnancy loss, flow cytometry, peripheral T helper cell, immune cell, NK cell, γδT cell

## Abstract

Recurrent pregnancy loss (RPL) is a disturbing disease in women, and 50% of RPL is reported to be associated with immune dysfunction. Most previous studies of RPL focused mainly on the relationship between RPL and either T cells or natural killer (NK) cells in peripheral blood and the decidua; few studies presented the systemic profiles of the peripheral immune cell subsets in RPL women. Herein, we simultaneously detected 63 immune cell phenotypes in the peripheral blood from nonpregnant women (NPW), women with a history of normal pregnancy (NP) and women with a history of RPL (RPL) by multi-parameter flow cytometry. The results demonstrated that the percentages of naïve CD4^+^ T cells, central memory CD4^+^ T cells, naïve CD8^+^ T cells, mature NK cells, Vδ1^+^ T cells and the ratio of Vδ1^+^ T cells/Vδ2^+^ T cells were significantly higher in the RPL group than those in the NPW and NP groups, whereas the percentages of terminal differentiated CD4^+^ T cells, effective memory CD4^+^ T cells, immature NK cells and Vδ2^+^ T cells were significantly lower in the RPL group than those in the NPW and NP groups. Interestingly, we found that peripheral T helper (T_PH_) cells were more abundant in the NPW group than in the NP and RPL groups. In addition, we also determined the 5^th^ percentile lower limit and 95^th^ percentile upper limit of the significantly changed immunological parameters based on the files of the NPW group. Taken together, this is the first study to simultaneously characterize the multiple immune cell subsets in the peripheral blood at a relatively large scale in RPL, which might provide a global readout of the immune status for clinicians to identify clinically-relevant immune disorders and guide them to make clear and individualized advice and treatment plans.

## Introduction

Recurrent pregnancy loss (RPL) is defined as two or more fetal losses and affects approximately 1% - 2% of reproductive women and < 5% of couples (1, 2). Several factors contribute to the pathogenesis of RPL, including chromosomal abnormalities, abnormal uterine anatomy, antiphospholipid antibodies, endocrine factors, infection and autoimmune disorders. However, nearly 50% of RPL cannot be explained by known causes, which are classified as unexplained RPL (3). Although the knowledge of unexplained RPL is limited, emerging studies have shown that unexplained RPL is associated with immunological factors, including peripheral and decidual immune states (4–8). During the menstrual cycle and pregnancy, immune cell subtypes, including natural killer (NK) cells, T cells and gamma-delta (γδ) T cells, in the peripheral and local microenvironments changed dynamically with the fluctuation and secretion of hormones (9, 10). A recent study by Han et al. (11) determined the dynamic alterations of over 370 immune cell features (including cell distribution and functional responses) in maternal blood of normal pregnancy and preeclampsia (PE) by using a high-dimensional mass cytometry immunoassay. They found differentially dynamics of maternal immune system between healthy and preeclamptic pregnancies, suggesting that the determination of the peripheral immune states might lay the groundwork for identifying clinically-relevant immune disorders in women with pathological pregnancy.

The establishment and maintenance of healthy pregnancy depend not only on immune homeostasis between the mother and the growing fetus from the blastocysts to the newborn stage, but also on the appropriate immune response of the maternal immune system (12). Unexplained RPL is still a major clinical challenge for gynecologists worldwide. Growing interest has focused on understanding the immunological mechanisms during pregnancy and how they are relate to pregnancy outcomes. A study has shown that uterine NK and T cells help to create immune tolerance and an embryo-compatible microenvironment to promote blastocyst implantation and invasion into the maternal decidua in early pregnancy, and abnormal uterine NK and T cells are involved in RPL (13). A reference range of uterine NK cell percentages in fertile women has been established by an immunochemistry assay with CD56 staining, and the percentages above and below the reference range may increase the risk of RPL (14). However, in this study, only major NK cells were detected; NK cell subtypes were not measured. Moreover, the detection of uterine NK cells is generally invasive and may be uncomfortable for women. Therefore, it is essential to determine the percentages of immune cell subsets in the peripheral blood and provide guidance for clinicians in the evaluation of RPL patients.

In recent years, emerging studies have focused on the relationship between lymphocytes in the peripheral blood and the pathogenesis of RPL. Several studies have shown that enhanced peripheral blood CD56^dim^ NK cells and abnormally high circulating NK cells expressing inhibitory cytokines and inhibitory surface receptors contributed to RPL (15–18). A meta-analysis indicated that the numbers of peripheral blood NK (pbNK) cells in infertile women were significantly higher than those in fertile women (19). Moreover, both the numbers and the percentages of pbNK cells in women with RPL were significantly increased compared with those in controls. However, the percentages of uNK cells were not significantly different in women with RPL compared with the controls. In addition, T cell immune balance is also associated with the onset of RPL. Decreased regulatory T (Treg) cells/exhausted T cells and increased Th17 cells/exhausted Treg cells were found in the peripheral blood of RPL patients by flow cytometry (20). A significantly increased Th17/Treg ratio in peripheral blood was observed in women with a history of RPL compared with those without the history of RPL (21, 22). Besides, the cytotoxicity of pbNK cells is negatively correlated with CD8^+^ T cells in normal pregnant women but not in women with the history of RPL (23). Although many prior studies have shown that immune cells in the peripheral blood are involved in RPL, they often focus on single or selected immune cell subsets, and are lack of a systemic and simultaneous assessment of multiple immune cell subsets. Thus, limited information is provided, and sometimes controversial results exist.

γδ T cells also constitute a major lymphocyte population in peripheral blood and constitute 15%-60% of peripheral blood mononuclear cells (PBMCs). γδ T cells are different from NK cells and T cells and known to bridge innate and adaptive immunity (4). Human γδ T cells are divided into two main subtypes according to the rearranged VA chain: Vδ1^+^ and Vδ2^+^ T cells. Vδ2^+^ T cells are distributed mainly in the peripheral blood and possess defense properties against pathogens, whereas Vδ1^+^ T cells are predominant in tissues such as the spleen, intestine, and liver and possess regulatory and effector properties (24). Talukdar et al. (6) reported that a significant decrease in the proportion of CD3^+^CD4^-^CD8^-^γδ T cells and an increase in the percentage of IFN-γ- and IL-17-producing γδ T cells in peripheral blood were associated with RPL by creating an inflammatory cytokine milieu. However, the relationship between Vδ1^+^ and Vδ2^+^ T cells and RPL remains unclear.

**Figure 1:**
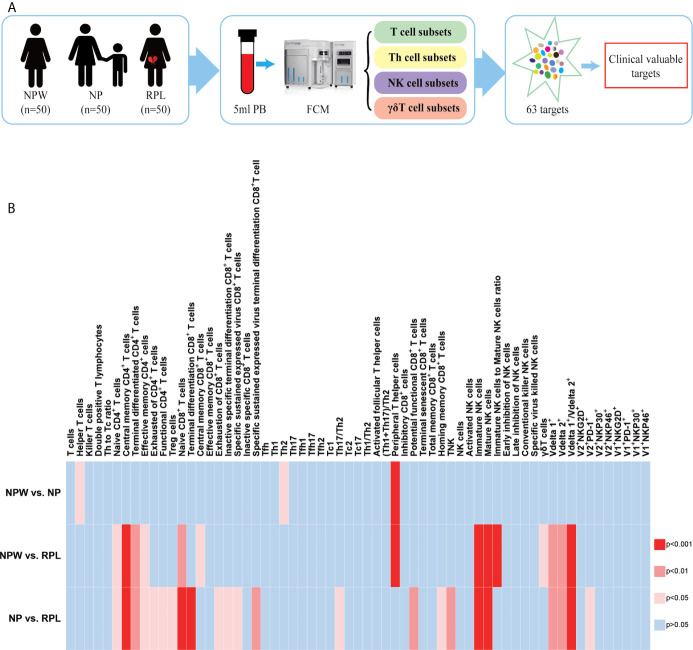
Study design and the overview of the different immunological parameters among the three groups. **(A)** Schedule of the study. Three groups were included in our study: NPW group (women who were never pregnant, n=50), NP group (women with the history of normal pregnancy, n=50) and RPL group (women with the history of RPL, n=50). Peripheral blood mononuclear cells (PBMCs) were isolated from 5 ml peripheral blood. Total 63 immune cell subsets were simultaneously detected by flow cytometry, including T cell, NK cell and γδ T cell subsets. By analyzing the data, clinical-relevant immune parameters were finally identified. **(B)** Heat map of the significantly changed immune parameters. The 63 immunological parameters were compared among the three groups and presented as the heat map, which can directly show the differences. The colors represent the different significance among the comparisons. The deeper the color is red, the larger the differences are. The blue represents no significance. NPW, women never pregnant; NP, women with a history of normal pregnancy; RPL, women with a history of RPL.

Significant scientific advances in immunological researches have shown that many immune cells participate in the pathogenesis of pregnancy complications; however, due to the diversity of the phenotype and function of immune cells, there is still lacking a simultaneously global readout of multiple immune cell subsets for the evaluation of RPL. Moreover, desperate RPL patients often ask for an “immune cell testing” or “immune therapy”, although there is insufficient evidence to support or ensure the diagnostics and treatment (25). Therefore, it is valuable to develop a set of immune cell panels to examine and evaluate immune cell subsets in RPL patients by noninvasive peripheral blood testing. Herein, we designed a cohort study in nonpregnant women (NPW), women with histories of normal pregnancy (NP) and RPL. The immunological profiles in NPW group were used as baseline controls, which are absent in most published studies. Five milliliters of peripheral blood were donated and analyzed by multiparameter flow cytometry, which can simultaneously detect 63 different immune cell subtypes. The study design and results are shown in **Figure 1**. The results showed that 10 kinds of immune cell subsets were specifically different in the RPL group and might be valuable for evaluating the potential risk of RPL. The 10 RPL-related immunological parameters included naïve CD4^+^ T cells (CD3^+^CD4^+^CD45RA^+^CCR7^+^), central memory CD4^+^ T cells (CD3^+^CD4^+^CD45RA^-^CCR7^+^), naïve CD8^+^ T cells (CD3^+^CD8^+^CD45RA^+^CCR7^+^), mature NK cells (CD3^-^ CD56^+lo^), Vδ1^+^ T cells (CD3^+^ γδ^+^Vδ2^-^ T), the ratio of Vδ1^+^ T cells/Vδ2^+^ T cells, terminal differentiated CD4^+^ T cells (CD3^+^ CD4^+^ CD45RA^+^ CCR7^-^), effective memory CD4^+^ T cells (CD3^+^CD4^+^CD45RA^-^CCR7^-^), immature NK cells (CD3^-^ CD56^+hi^) and Vδ2^+^ T cells (CD3^+^ γδ^+^Vδ2^+^ T); the first 6 were found at higher levels and the latter 4 at lower levels in the RPL group than in both the NPW and NP groups. Interestingly, we also found that the percentage of T_PH_ cells (CD3^+^CD4^+^CXCR5^-^PD-1^+^) in the NPW group was higher than that in both the NP and RPL groups. To further analyze these data, we measured the 5^th^ percentile limit and 95^th^ percentile limit of these significantly different parameters to set up the reference ranges for diagnosing, predicting and evaluating the risk of RPL and therapeutic efficacy in future; levels above the 95^th^ percentile limit and below the 5^th^ percentile limit indicate an increased risk of RPL, which might lay the groundwork for clinicians to identify clinically-relevant immune disorders and create clear and individualized therapeutic plans for RPL patients.

## Materials and Methods

### Subjects

Three groups were included in our study: women without any pregnancy (NPW), women with the history of normal pregnancy (NP) and women with the history of RPL (RPL). No participants in the NPW group smoked or consumed alcohol excessively, and all had regular menstrual cycles and were considered to be healthy. In the NP group, each woman delivered a healthy baby within 1-2 years and had no history of abortion or pathological pregnancy. All the women were in good health with regular menstrual cycles. The women in the RPL group experienced two or more pregnancy losses before 20 weeks of gestation and did not exhibit chromosomal abnormalities, abnormal uterine anatomy, antiphospholipid antibodies, endocrine factors, infection and autoimmune disorders.

This study was performed at the Center for Reproductive Medicine at Tongji Medical College of Huazhong University of Science and Technology (HUST), Wuhan, China from April 2018 to August 2019. The protocol was reviewed and approved by the Clinical Trial Ethics Committee of HUST (2018S392). All methods were carried out in accordance with the approved guidelines and regulations. Written informed consent was obtained from all the study subjects prior to entering the study.

### Multiple Parameter Flow Cytometry

**Figure 2:**
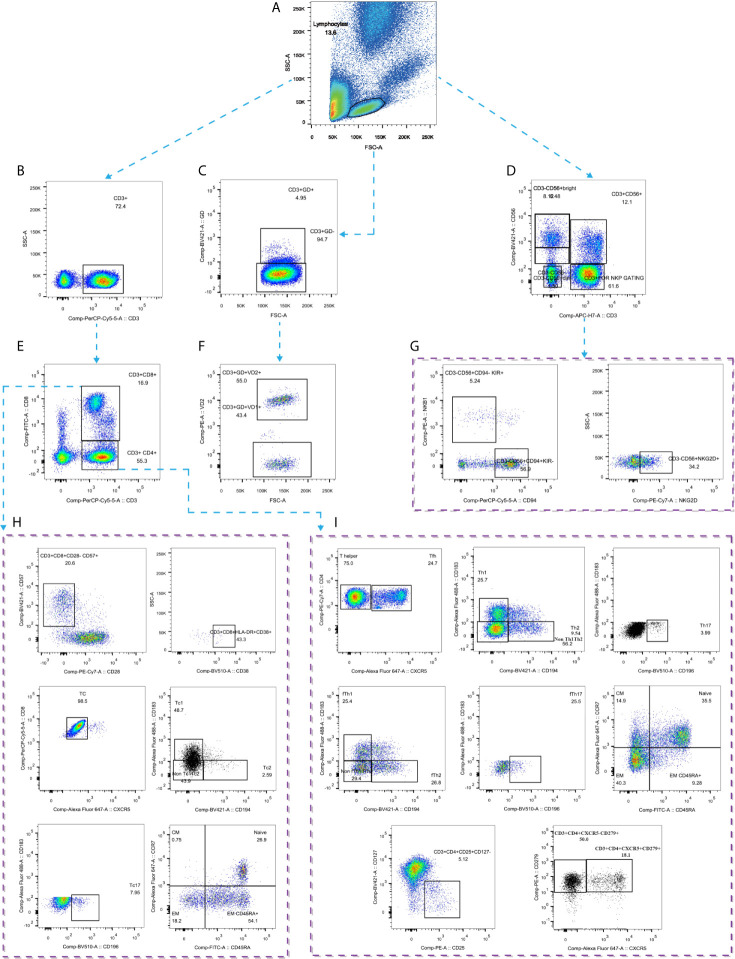
Gating strategies by flow cytometry. **(A)** Gating strategy for lymphocytes; **(B)** Gating strategy for CD3^+^ T cells; **(C)** Gating strategy for CD3^+^γδ^+^ T cells; **(D)** Gating strategy for CD3^-^CD56^+^ NK cells; **(E)** Gating strategy for CD4^+^ and CD8^+^ T cells; **(F)** Gating strategy for Vδ1^+^ and Vδ2^+^ T cells; **(G)** Gating strategy for NK cell subtypes; **(H)** Gating strategy for CD8^+^ T cells subtypes; **(I)** Gating strategy for CD4^+^ T cell subtypes. GD: γδ.

Five milliliters of peripheral blood were collected in heparin-treated tubes from each individual in the three groups and sent for detection within 24 h. Blood samples were diluted 1:1 with PBS buffer after plasma selection and then lightly laid on Ficoll-Hypaque media (Pharmacia). PBMCs were isolated by density gradient centrifugation. Cells were washed in RPMI 1640 supplemented with 10% FBS (Gibco) and then used immediately for multiparametric flow cytometry. The experiments were performed according to the manufacturer’s instructions. Flow cytometry was performed on a BD LSRFortessa X-20, and the data were analyzed with FlowJo V10 software (Tree Star). The gating strategy is shown in **Figure 2**.

### Statistics

The Shapiro-Wilk test was used to check the data distribution. For the normally distributed data, the results are shown as the mean with standard deviation (SD). Median and range were used for skewed data. The 5^th^ and 95^th^ percentiles of the immunological parameters represented the lower and upper limits for the reference ranges. Ordinary one-way ANOVA (multiple comparisons) was used to determine significant differences among the three groups. Significance levels were set to *P < 0.05, **P < 0.01, ***P < 0.001 and ****P < 0.0001, and *ns* means not significant. All statistical data were analyzed using GraphPad Prism version 7.0.

## Results

### Demographics

All the subjects were matched with the inclusion and exclusion criteria. The median age of the three groups was 24 years in the NPW group (range: 23-25 y), 31 years in the NP group (range: 28-33 y) and 30 years in the RPL group (range: 28-32 y). The median BMI of the three groups was 20.12 in the NPW group (range: 19.25-21.58), 21.21 in the NP group (range: 19.42-23.55) and 21.5 in the RPL group (range: 19.10-22.90). The median gravidity of women in the NP group was 1, whereas that of women in the RPL group was 2. The women in the NP group all delivered 1 healthy baby (**Table S1**).

### Significantly Changed Immune Parameters Related to RPL

**Figure 3:**
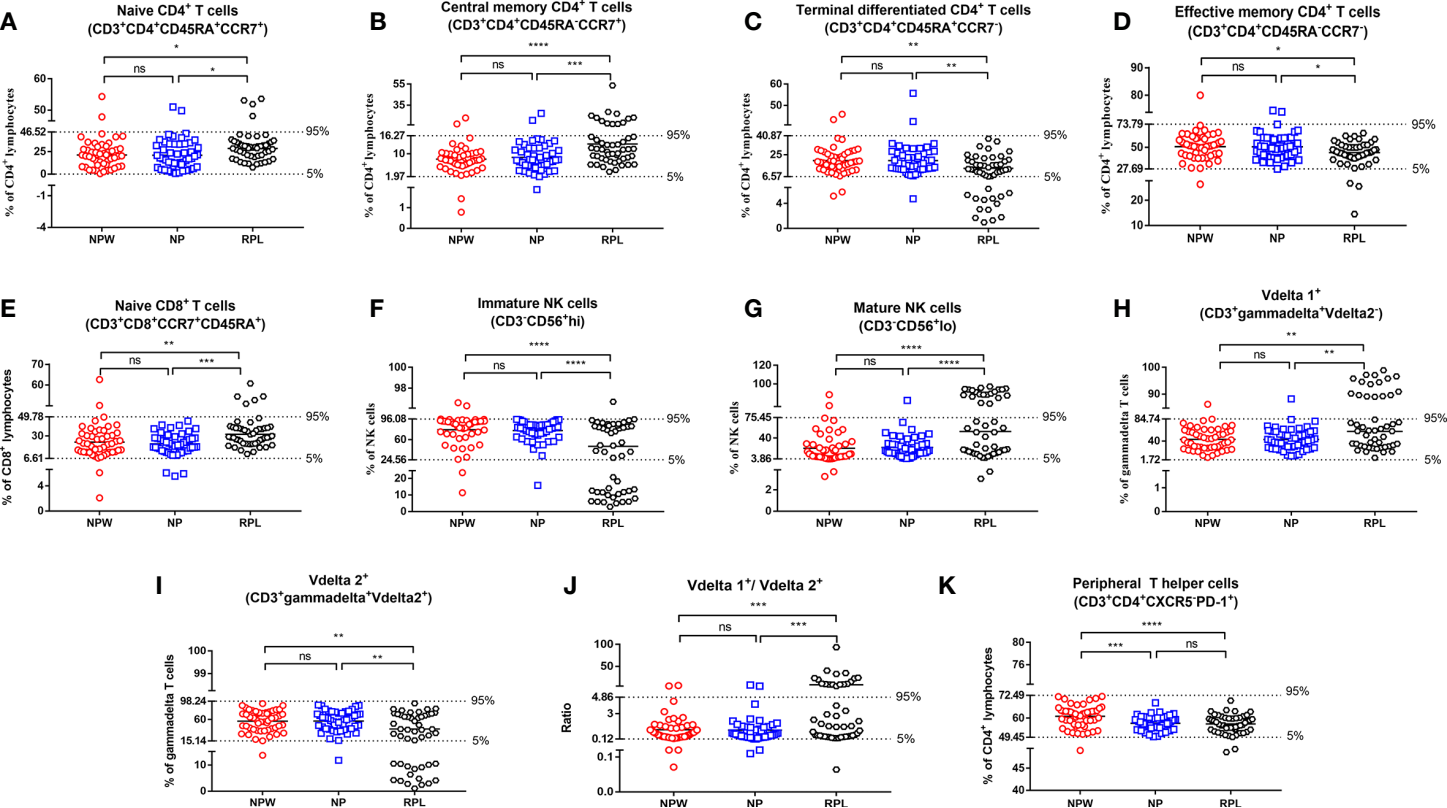
Reference ranges for the significantly changed immune parameters. **(A–J)** Reference ranges for significantly changed immune parameters related to RPL. The percentages of naïve CD4^+^ T cells **(A)**, central memory CD4^+^ T cells **(B)**, naïve CD8^+^ T cells **(E)**, mature NK cells **(G)**, Vδ1^+^ T cells **(H)** and the ratio of Vδ1^+^ T cells/Vδ2^+^ T cells **(J)** were significantly higher in the RPL group than those in the NPW and NP groups. The percentages of terminal differentiated CD4^+^ T cells **(C)**, effective memory CD4^+^ T cells **(D)**, immature NK cells **(F)** and Vδ2^+^ T cells **(I)** were significantly lower in the RPL group than those in the NPW and NP groups. The mean ± 1.96 SD was used to measure the reference ranges for the normally distributed data. Median and 5^th^/95^th^ percentiles represented the lower/upper limit to set up the reference ranges for skewed distribution data. In 14%- 40% of women with RPL, the percentages of central memory CD4^+^ T cells **(B)**, mature NK cells **(G)**, the ratio of Vδ1^+^ T cells/Vδ2^+^ T cells **(J)**, and Vδ1^+^ T cells **(H)** were above the 95^th^ percentile limit. In 28% - 40% of women with RPL group, the percentages of terminally differentiated CD4^+^ T cells **(C)**, immature NK cells **(F)** and Vδ2^+^ T cells **(I)** were below the 5^th^ percentile limit. The percentages of these different immunological parameters in the NP group were similar to those in the NPW group, and most were within the 5^th^ percentile limit and 95^th^ percentile limit. **(K)** Reference ranges for significantly changed immune parameters related to pregnancies. The percentage of T_PH_ was significantly low in the NP and RPL groups compared with the NPW group. Vdelta1: Vδ1; Vdelta2: Vδ2. Significance levels were set to *P < 0.05, **P < 0.01, ***P < 0.001, and ****P < 0.0001, and *ns* means not significant.

We collected 5 ml of peripheral blood from 50 participants in each group and simultaneously measured 63 immune cell subsets (**Figure 1A**). The overall alternation of all immunological parameters among the three groups was shown in **Figure 1B**. Previous studies reported that CD4^+^ T cells biased to Th2 and/or Treg cells, focusing mainly on the Th1/Th2 and Th17/Treg balance, promote the development of normal pregnancy (26–28). Although recent advances in reproductive immunology have expanded the Th1/Th2/Th17/Treg paradigm to the Th1/Th2/Th9/Th17/Th22/follicular T helper (Tfh) paradigm (29), this could not uncover the whole knowledge of the immunological factors in RPL. The profiles of other T cell subtypes in the immunological etiology of RPL should also be worthy investigation. Here, we analyzed the percentages of CD3^+^CD4^+^ and CD3^+^CD8^+^ T cell subtypes in T cell development to determine the immunological factors involved in RPL. The results showed that there were no significant differences in the percentages of Th1 (CD3^+^CD4^+^CXCR5^-^CXCR3^+^CCR4^+^), Th2 (CD3^+^CD4^+^CXCR5^-^CXCR3^-^CCR4^+^), Th17 (CD3^+^ CD4^+^CXCR5^-^CXCR3^-^CCR4^-^CCR6^+^) and Treg cells (CD3^+^CD4^+^ CD25^+^CD127^-^) (**Figures S1Aa–d**) among 3 groups, indicating that these immune cell subsets might be not related to RPL in a large population. Interestingly, we found that 5 novel T cell subsets were significantly different in the RPL group compared with the NPW and NP groups, which have never been reported before in RPL. They are naïve CD4^+^ T cells (CD3^+^CD4^+^CD45RA^+^CCR7^+^) (*P* < 0.05) (**Figure 3A**), central memory CD4^+^ T cells (CD3^+^CD4^+^CD45RA^-^CCR7^+^) (*P* < 0.001) (**Figure 3B**), terminal differentiated CD4^+^ T cells (CD3^+^ CD4^+^ CD45RA^+^ CCR7^-^) (*P* < 0.01) (**Figure 3C**), effective memory CD4^+^ T cells (CD3^+^CD4^+^CD45RA^-^CCR7^-^) (*P* < 0.05) (**Figure 3D**) and naïve CD8^+^ T cells (CD3^+^CD8^+^CD45RA^+^CCR7^+^) (*P* < 0.01) (**Figure 3E**). In the screening of 41 immunological parameters of T cells, most of them were not significantly different in RPL group compared with NPW and NP groups, including Th cell subtypes (**Figure S1A**), CD8^+^ T cell subtypes (**Figure S1B**), CD4^+^ T cell subtypes (**Figure S1C**), Tfh cells (**Figure S1D**) and their ratios (**Figure S1E**). In addition, the percentage of T_PH_ cells (CD3^+^CD4^+^CXCR5^-^PD-1^+^) (**Figure 3K**) was significantly lower in the NP (*P* < 0.001) and RPL (*P* < 0.0001) groups than that in the NPW group.

Besides, we also detected peripheral NK cell subtypes, which consist of approximately 10-15% circulating lymphocytes and are associated with the innate immune response. Among 10 kinds of NK cell subtypes, the percentage of CD3^-^CD56^+hi^ cells (immature NK cells) (*P* < 0.0001) (**Figure 3F**) was significantly lower in the RPL group than that in the NPW and NP groups, whereas the percentage of CD3^-^CD56^+lo^ cells (mature NK cells) (*P* < 0.0001) (**Figure 3G**) was significantly higher in the RPL group than that in the NPW and NP groups. Another 8 kinds of NK cell subtypes were not significantly different among the three groups (**Figure S2**).

γδ T cells are a special immune cell subset and different from T cells in their T cell receptor (TCR) chains. γδ T cells can link innate and adaptive immune responses (30). Among 12 kinds of γδ T cell subtypes, three were statistically changed in the RPL group. The percentage of Vδ1^+^ T cells (CD3^+^ γδ^+^Vδ2^-^ T) (*P* < 0.01) (**Figure 3H**) and the ratio of Vδ1^+^ T cells/Vδ2^+^ T cells (*P* < 0.001) (**Figure 3J**) in the RPL group were higher than those in both the NPW and NP groups. However, the percentage of CD3^+^ γδ^+^Vδ2^+^ T (Vδ2^+^ T cells) (*P* < 0.01) (**Figure 3I**) was significantly lower in the RPL group than that in the NPW and NP groups. The percentage of other γδ T cell phenotypes were not significantly different among the three groups (**Figure S3**).

### Detailed Profiles in the Percentages of the Significantly Changed Immune Parameters

We further described the detailed percentages of the total 11 immunological parameters with significant differences either in the mean and SD, or the median and range. The mean percentages of naïve CD4^+^ T cells and effective memory CD4^+^ T cells were 21.12% (SD=12.96%) and 50.74% (SD=11.76%), respectively. The median percentages of central memory CD4^+^ T cells, terminal differentiated CD4^+^ T cells and naïve CD8^+^ T cells were 7.83% (range: 5.89% - 9.96%), 18.70% (range: 13.35%-25.95%) and 21.55% (range: 13.33%-32.53%), respectively. For NK cells and γδ T cells, the median percentages of immature NK cells, mature NK cells and the ratio of Vδ1^+^ T cells/Vδ2^+^ T cells were 87.35% (range: 69.80%-92.23%), 12.35% (range: 7.07%-29.63%) and 0.74 (range: 0.35-1.56), respectively. The mean percentages of Vδ1^+^ and Vδ2^+^ T cells were 43.23% (SD= 21.18%) and 56.69% (SD=21.2%), respectively. The mean percentage of T_PH_ cells was 60.97% (SD=5.88%) (**Table 1**).

**Table 1:** Characteristics of 11 immune parameters with significant differences in NPW, NP and RPL groups.

	Mean/Median	SD/Range	95% upper limit	5% lower limit	NPW			NP			RPL		
					Higher (%)	In (%)	Lower (%)	Higher (%)	In (%)	Lower (%)	Higher (%)	In (%)	Lower (%)
Naïve CD4^+^ T cells^a^	21.12	12.96	46.52	-4.28	4	96	0	4	96	0	8	92	0
Central memory CD4^+^ T cells^b^	7.83	5.88-9.96	16.27	1.97	4	92	4	4	94	2	24	76	0
Terminal different CD4^+^ T cells^b^	18.70	13.35-25.95	40.87	6.57	4	92	4	4	94	2	0	72	28
Effective memory CD4^+^ T cells^a^	50.74	11.76	73.79	27.69	2	96	2	4	96	0	0	94	6
Naïve CD8^+^ T cells^b^	21.55	13.33-32.53	49.78	6.61	4	92	4	0	94	6	10	90	0
Immature NK cell^b^	87.35	69.80-92.23	96.08	24.56	4	92	4	0	98	2	2	58	40
Mature NK cells^b^	12.35	7.07-29.63	75.45	3.86	4	92	4	2	98	0	40	56	4
Vdelta 1^+ a^	43.23	21.18	84.74	1.72	2	98	0	2	98	0	32	68	0
Vdelta 2^+ a^	56.69	21.2	98.24	15.14	0	98	2	0	98	2	0	68	32
Vdelta 1^+^/Vdelta 2^+b^	0.74	0.35-1.56	4.86	0.12	4	94	2	4	94	2	34	64	2
Peripheral T helper cells^b^	60.97	5.88	72.49	49.45	0	98	2	0	100	0	0	96	4

^a^mean/SD; ^b^median/range.

### Reference Value Ranges of the Significantly Changed Immune Parameters

Although previous studies reported that abnormal NK cells, T cells and γδ T cells in the peripheral blood were involved in the pathogenesis of RPL, there is lacking consistent reference value ranges for the clinicians to identify the clinically-relevant immune dysfunctions. In the current study, we used the files of NPW as the baseline to fill this gap. Basing on the data distribution, we applied the mean ± 1.96 SD and 5^th^ and 95^th^ percentiles to set up the reference value ranges of the different parameters. For the RPL-related indicators, the reference ranges (5^th^ lower limits and 95^th^ upper limits) for naïve CD4^+^ T cells, central memory CD4^+^ T cells, terminal differentiated CD4^+^ T cells, effective memory CD4^+^T cells, naïve CD8^+^ T cells, immature NK cells, mature NK cells, Vδ1^+^ T cells, Vδ2^+^ T cells and the ratio of Vδ1^+^ T cells/Vδ2^+^ T cells were 0-46.52%, 1.97%-16.27%, 6.57%-40.87%, 27.69%-73.79%, 6.61%-49.78%, 24.56%-96.08%, 3.86%-75.45%, 1.72%-84.74%, 15.14%-98.24%, and 0.12-4.86, respectively. The reference range of T_PH_ cells was 49.45%-72.49%. Only 0-4% of the NPW exhibited values outside of the reference ranges (**Table 1**).

### Distribution Characteristics of Significantly Changed Immune Parameters

The reference ranges derived from NPW were used to assess the distribution of the different immunological parameters in women with NP or RPL. In most women with NP, the values were within the reference ranges, and only 0-6% (0-3) of women were not in the reference ranges. However, more fluctuation occurred in the significantly elevated and decreased immunological parameters in the RPL group. Specifically, among the elevated immunological parameters, the values above the reference ranges in the RPL group were 24% in central memory CD4^+^ T cells, 40% in mature NK cells, 32% in Vδ1^+^ T cells and 34% in Vδ1^+^ T cells/Vδ2^+^ T cells, and the values below the 5^th^ percentile limit in the RPL group were 28% in terminal differentiated CD4^+^ T cells, 40% in immature NK cells and 32% in Vδ2^+^ T cells. All recruited women in the NP group exhibited a level of T_PH_ cells within the reference ranges, whereas 4% of women with RPL exhibited values outside the reference range (**Table 1**).

### Immunological Panel

In this study, we simultaneously detected 63 immune cell subsets in the peripheral blood from NPW, NP and RPL groups and identified 11 statistically significant parameters. Basing on the immune surface markers and functional molecules of the immune cell subtypes, we designed a potential immunological panel for evaluating the risk of women with RPL: CD3/CD4/CD8/CD56/CD45RA/CCR7/γδ T/Vδ2/CXCR5/PD-1. This panel might be used to identify the clinically-relevant immune disorders in PRL patients.

## Discussion

In this study, we simultaneously detected 63 immune cell subsets in the peripheral blood of women without a history of pregnancy and those with histories of NP or RPL, including T cell, NK cell and γδT cell subsets. Also, we defined the reference ranges for each significantly changed parameters. Most prior studies focused on single or selected immune cells, such as T cells and NK cells, in the pathogenesis of RPL (4, 27, 31). In our study, we not only detected the major immune cell subsets as previous studies reported, but also examined the immune cells at different developmental stages, and the special activating and inhibitory receptors and the specific functional molecules expressed on these cells (**Table 2**). Comparing with the NPW and NP groups, 10 significantly high or low immunological parameters in RPL group were found. Moreover, the percentage of T_PH_ cells was significantly lower in the NP and RPL groups than that in the NPW group. To our knowledge, this is the first study to simultaneously detect multiple immunological parameters and reveal their hallmarks in RPL, which might provide new insights and individualized evaluation for clinicians to identify clinical-relevant immune dysfunctions in RPL patients.

**Table 2:** Potential functions of significantly different immune cell subsets.

Cell subsets	Potential functions	Ref
Naïve CD4^+^ T cells	Naïve CD4^+^ T cells differentiate to effector T cells and subsequently develop into long-lived memory T cells.	(32, 33)
Naïve CD8^+^ T cells	Naïve CD8^+^ T cells differentiate to effector T cells and subsequently develop into long-lived memory T cells.	(32, 33)
Terminal differentiated CD4^+^ T cells	Terminally differentiated CD4^+^ T cells are associated with protection, though they do not have the ability of renewal and differentiation.	(34)
Central memory CD4^+^ T cells	Central memory CD4^+^ T cells mediate reactive memory, readily proliferate, and differentiate to effector cells and produce large amounts of IFN-γ or IL-4 in response to antigenic stimulation.	(35, 36)
Effective memory CD4^+^ T cells	Effective memory CD4^+^ T cells mediate protective memory, display immediate effector function.	(35, 36)
Immature NK cells	Immature NK cells have low cytotoxicity and high production of cytokines and chemokines, including M-CSF and GM-CSF.	(37)
Mature NK cells	Mature NK cells are functionally well known for their potent cytotoxic activity.	(37–39)
Vδ1^+^ T cells	Vδ1^+^ T cells possess both regulatory and effector properties. Vδ1 T cells could kill tumor cells and have pro-inflammatory properties.	(40–42)
Vδ2^+^ T cells	Vδ2^+^ T cells exert a cytolytic effect against pathogenic properties.	(40)
Peripheral T helper cells (T_PH_)	T_PH_ cells are uniquely antigen-specific T cells with increased expression of genes associated with B cell functions.	(43–45)

In our study, peripheral T cell subsets were assessed. The percentages of naïve CD4^+^ T cells, naïve CD8^+^ T cells, and central memory CD4^+^ T cells were significantly increased in RPL. However, the percentages of terminal differentiated CD4^+^ T cells and effective memory CD4^+^ T cells were significantly decreased in RPL. In the development of T cells, primary T cells undergo TCR rearrangement from bone marrow (BM) progenitors lacking CD4^+^ and CD8^+^ coreceptors to generate CD4^+^CD8^+^ double-positive (DP) T cells and then undergo single selection to give rise to CD4^+^ or CD8^+^ single-positive (SP) cells. Ultimately, SP T cells enter the periphery as naïve T cells with CD45RA^+^CCR7^+^ phenotypes (46). When naïve T cells encounter antigen and costimulatory ligands presented by dendritic cells (DCs), they proliferate and differentiate into effector T cells. Naïve T cells can regulate T cell antigen signaling and further differentiate into effector and memory T cells (32, 33). Activated effector T cells are short-lived, and only small proportions survive as memory T cells that persist as heterogeneous subsets (47). Memory T cells are characterized by CD45RA^-^ (48), whereas effector T cells are by CCR7^-^. Effective memory T cells and terminal effector T cells, which are present in the circulation, exhibit CD45RA^-^CCR7^-^ and CD45RA^+^CCR7^-^ phenotypes, respectively (49, 50).

Compared with pre-pregnancy endometrium, the proportion of naïve T cells in the decidua of full-term pregnancy was significantly reduced, whereas that of memory T cells was significantly increased (51). Memory is the hallmark of the acquired immune system and results from clonal expansion and differentiation of antigen-specific lymphocytes that ultimately persist for a lifetime (35, 52). Effective memory T cells migrate to inflamed peripheral tissues and display immediate effector function, which mediate protective memory, whereas central memory T cells are homing to T cell areas of secondary lymphoid organs and rapidly proliferate and differentiate into effector cells in response to antigenic stimulation, mediating reactive memory (35). Previous study found that effective memory CD4^+^ T cells were increased in normal pregnancy (36). Central memory CD4^+^ T cells were significantly increased in the peripheral blood of preeclamptic women and RPL women compared with the controls (36, 53). It was reported that terminally differentiated CD4^+^ T cells are associated with protection (34), whereas terminally differentiated CD8^+^ T cells play roles in persistent infections, especially cytomegalovirus (CMV) infection (49). In our study, the percentages of both terminal differentiated CD4^+^ T cells and terminal differentiated CD8^+^ T cells were lower in the RPL group than those in the NP group, suggesting that women with RPL might have low immune protection and be susceptible to infections. CD28, as a costimulatory molecule, is mainly expressed in CD8^+^ T cells at birth, and its downregulation is a hallmark of senescence and exhaustion (54). We found that the percentage of exhaustion of CD8^+^ T cells (CD8^+^ CD28^-^ T cells, immunosuppressive phenotype) was significantly lower, whereas that of potential functional CD8^+^ T cells (CD8^+^ CD28^+^ T cells) were higher in RPL group than in NP group. The results indicated that the immune system in women with a history of RPL might be in a less immunosuppressive state.

Peripheral NK cells consist of approximately 10-15% circulating lymphocytes. Basing on the expression of CD56, NK cells are classically divided into CD3^-^ CD56^bright^ (high CD56 intensity) and CD3^-^ CD56^dim^ (low CD56 intensity) (55). NK cells belong to the innate immune system and are widely distributed in human tissues. A high frequency of NK cells was found in the circulation, lungs, liver, and uterus (56). CD3^-^ CD56^+^ NK cells can be differentiated from CD34^+^ common lymphocyte progenitors (CLPs) with CD34 downregulation and CD56 upregulation. CD94 helps the development of CD56^bright^ NK cells, whereas CD56^bright^ NK cells subsequently differentiate into CD56^dim^ NK cells with upregulation of CD16 (57). CD3^-^CD56^dim^ NK cells are the predominant subset of pbNK cells and account for approximately 90% of total pbNK cells, which are functionally well known for their potent cytotoxic activity and are marked as CD3^-^CD56^dim^CD16^+^ NK cells (58). In line with the previous studies, CD3^-^CD56^dim^CD16^+^ NK cells possess high cytolytic activity and were increased in RPL, which might be involved in the demise of the conceptus (38, 39). In contrast, CD3^-^ CD56^bright^ NK cells account for only 10% of pbNK cells with low cytotoxicity and high production of cytokines and chemokines (59). A previous study showed that decreased peripheral CD3^-^CD56^bright^ NK cells in RPL were associated with the altered cytokine expression profile (37). Our findings further confirmed that CD3^-^CD56^+hi^ NK cells with lower cytotoxicity were significantly lower in the RPL group compared to that in the NPW and NP groups. In addition, many molecules are expressed on NK cells, including natural cytotoxicity receptors, inhibitory receptors, immune checkpoints and inhibitory molecules (60). NK cell functions are also closely associated with the expression of activating and killing inhibitory receptors (KIRs). KIRs can interact with neighboring receptors, such as activating receptors - natural killer group 2, member D (NKG2D), natural cytotoxicity receptors (NCRs) NKp30 (also known as NCR3), NKp44 (also known as NCR2) and NKp46 (also known as NCR1) (61, 62) and KIRs – CD94/NKG2A (KIRD1/CD159a) heterodimers, which belong to HLA-class I (HLA-cl I)-specific inhibitory receptors (60). In our study, the NK cell phenotypes with specific functional molecules were not statistically different among the three groups. These data indicated that NK cells with high cytotoxicity were more enriched than NK cells with low cytotoxicity in RPL, which might be related to RPL. However, there were no differences in the percentages of activated NK cells, early and late inhibitory NK cells, conventional killer NK cells or specific virus-killed NK cells among RPL, NP and NPW groups (**Figures S2A–G**).

Unlike αβ T cells and NK cells, γδ T cells share characteristics of innate and adaptive immune cells (63). γδ T cells represent 3–5% of total T cells in the peripheral blood and can be classified into two main populations, Vδ1 and Vδ2 T cells, according to their TCR variable (V) gene segment usage. Human Vδ2 T cells are the main γδ T cell subtypes in the peripheral blood (64). However, Vδ1 T cells are mainly distributed in epithelial tissues (65). γδ T cells can recognize cells under stressed conditions, particularly infected or transformed cells, and kill them or regulate the immune response against them, paving the way for the development of promising therapeutic strategies for cancer and infectious diseases (66). γδ T cells employ T cell receptors and/or NK cell receptors, including major histocompatibility complex (MHC) molecules and NK cytotoxicity receptors (NCRs) on their surface (such as NKG2D, NKp44 and NKp30), for target cell recognition, which accounts for their remarkable flexibility (67–69). In the maternal-fetal interface, the majority of γδT cells were Vδ1 and produce high levels of TGF-β and IL-10, which might be a key player in maintaining the Th2 bias. Aberrant increase of decidual Vδ2 T cells in early-pregnancy is associated with unexplained spontaneous abortion (40). However, the function of γδT cells is strongly plastic. Previous studies have shown that Vδ1 T cells could efficiently kill myeloma cells and multiple epithelial tumor cell lines, with no significant difference in the specific lysis compared with Vδ2 T cells (70). Vδ1 T cells also have pro-inflammatory properties (41, 42). Interestingly, we found that the percentage of Vδ1^+^ T cells was significantly higher and that of Vδ2^+^ T cells was significantly lower in the RPL group than that in the NPW and NP groups. Additionally, the ratio of Vδ1^+^/Vδ2^+^ T cells was higher in the RPL group. Thus, it is necessary to further elucidate the function of γδT cell subsets in the peripheral blood and maternal-fetal interface of normal pregnancy and pregnancy related diseases including RPL.

In the current study, we also found that the percentage of T_PH_ cells was significantly higher in the NPW group than that in both the NP and RPL groups. T_PH_ cells are uniquely antigen-specific T cells with increased expression of genes including IL21 and CXCL13, which play important roles in B cell differentiation and potent B cell chemoattractants, respectively, and thus benefit the B cell function (43–45). In rheumatoid arthritis patients, T_PH_ cells were expanded in the active phage and decreased with effective treatment. Also, T_PH_ cells can be expanded in autoantibody-producing diseases (RA, SLE, SSc and coeliac disease), but not in diseases without autoantibodies (seronegative RA and spondyloarthritis), which indicate that T_PH_ cells augment B cell activation and autoantibody production (71). A previous study showed that IL-10-producing B cells were significantly low, whereas plasma cells were high in women with recurrent implantation failure (RIF). Since IL-10-producing B cells can suppress the production of autoantibodies and contribute to a successful implantation, their downregulation might be related to the occurrence of RIF (72). In this study, we did not detect the B cell populations and T_PH_ cells in women with RIF, which might limit the understanding of T_PH_ cells in implantation and needs further investigation.

To the best of our knowledge, this is the first observed finding; the detailed role and underlying mechanism in RPL require further investigations.

## Conclusions

Prior studies primarily focused on the assessment of single or selected peripheral immune cell subsets in RPL, which lacks the simultaneous and comprehensive evaluation of immunological profiles in women of childbearing age. Herein, we simultaneously read out the multiple immunological parameters in the peripheral blood of women without any pregnancy (NPW group), or with histories of NP or RPL. A set of 11 statistically changed immunological parameters were found, including naïve CD4^+^ T cells, naïve CD8^+^ T cells, central memory CD4^+^ T cells, terminal differentiated CD4^+^ T cells, effective memory CD8^+^ T cells, immature NK cells, mature NK cells, Vδ1^+^ T cells, Vδ2^+^ T cells, the ratio of Vδ1^+^ T cells/Vδ2^+^ T cells and T_PH_ cells. Many of these cell types are novel immune cell subtypes involved in pregnancy. Moreover, we also defined the reference ranges based on the 5^th^ percentile limit and 95^th^ percentile limit of each immunological parameter to guide clinicians in globally evaluating the immune status of women before pregnancy. Some immune cell subtypes in women with RPL (up to 48%) were above the 95^th^ percentile limit, whereas some were below the 5^th^ percentile limit. These novel immunological parameters related to RPL may provide new insight into the immunological pathogenesis of RPL. Nevertheless, there is a limitation. The current study cannot confirm whether the differences in levels of some immune cells are a cause or consequence of RPL. Thus, a prospective cohort study is required to prove the relationship between the significantly changed immune cell subsets and the pregnancy outcomes in RPL.

## Data Availability Statement

The original contributions presented in the study are included in the article/**Supplementary Material**. Further inquiries can be directed to the corresponding author.

## Ethics Statement

The studies involving human participants were reviewed and approved by the Clinical Trial Ethics Committee of Huazhong University of Science and Technology (2018S392). The patients/participants provided their written informed consent to participate in this study.

## Author Contributions

HL, X-XL, and X-BH were responsible for writing the manuscript, collecting samples, conception, and data analyzing. D-HH, SS, Y-JC, and JT were responsible for clinical sample collecting. DT, Z-NY, and GM were responsible for data analysis and interpretation. A-HL was responsible for data analysis, assembly and interpretation, study design and administration and revision. All authors contributed to the article and approved the submitted version.

## Funding

This work was supported by National Key Research & Developmental Program of China (2018YFC1003900; 2018YFC1003904).

## Conflict of Interest

The authors declare that the research was conducted in the absence of any commercial or financial relationships that could be construed as a potential conflict of interest.

## Supplementary Material

Supplementary Figure 1 Reference ranges for nonsignificant immune parameters in CD4^+^ and CD8^+^ T cells. **(A)** Th1 and Th17 cells were not significantly different in the RPL *vs.* NP and RPL *vs.* NPW groups. Th2 cells were significantly different in the NP *vs.* NPW groups but not in the RPL *vs.* NP and RPL *vs.* NPW groups. Treg cells were significantly different in the RPL *vs.* NP groups but not in the RPL *vs.* NPW groups. **(B)** T cells (a), killer T cells (b), effective memory CD8^+^ T cells (e), inactive specific CD8^+^ T cells (i), inhibitory CD8^+^ T cells (k), terminally senescent CD8^+^ T cells (m) and total memory CD8^+^ T cells (n) were not significantly different among the three groups. Some of the parameters were lower or higher than the 5^th^/95^th^ percentile limits. Terminal differentiation CD8^+^ T cells (c), exhaustion of CD8^+^ T cells (f), inactive specific terminal differentiation CD8^+^ T cells (g), specific sustained expressed virus CD8^+^ T cells (h), specific sustained expressed virus terminal differentiation CD8^+^ T cells (j), potential functional CD8^+^ T cells (l), and homing memory CD8^+^ T cells (o) were significantly different in the RPL *vs.* NP groups but not in the RPL *vs.* NPW groups. Central memory CD8^+^ T cells (d) were significantly different in the NPW *vs.* RPL groups but not in RPL *vs.* NP groups. **(C)** CD4^+^ T cell DP T lymphocytes and activated Tfh cells were not significantly different among the three groups. Helper T cells were significantly different in the NP *vs.* NPW groups. The exhaustion of CD4^+^ T cells and functional CD4^+^ T cells was significant in the RPL *vs.* NP groups but not in the RPL *vs.* NPW groups. **(D)** Tfh cells (a) and cytotoxic T (Tc) cells (Tfh, Tfh1, Tfh2, Tfh17, Tc1, Tc2 and Tc17) (b–g) were not significantly different among the three groups. **(E)** The Th to Tc ratios (a), Th1/Th2 ratios (c) and (Th1+Th17)/Th2 ratios (d) were nonsense parameters among the three groups. Th17/Th2(b) was significantly different in the RPL *vs.* NP groups. The ratio of immature NK cells to mature NK cells (e) was significantly different in the RPL *vs.* NPW groups. The mean ± 1.96 SD was used to measure the reference range for the normally distributed data. The median and 5^th^/95^th^ percentiles represent the lower/upper limits of each reference range for skewed distribution data. Significance levels were set to **P* < 0.05, ***P* < 0.01, and ****P* < 0.001 and *ns* means not significant.

Supplementary Figure 2 Reference ranges for nonsignificant immune parameters in NK cells. **(A–G)** TNK cells, NK cells, activated NK cells, early inhibition of NK cells, late inhibition of NK cells, conventional killer NK cells and specific virus killed NK cells were not significantly different among the three groups. The mean ± 1.96 SD was used to measure the reference range for the normally distributed data. The median and 5^th^/95^th^ percentiles represent the lower/upper limits of the reference ranges for skewed distribution data. Significance levels were set to *P* < 0.05, and *ns* means not significant.

Supplementary Figure 3 Reference ranges for nonsignificant immune parameters in γδ T cells. **(A)** γδ T cells were significantly different in the RPL *vs.* NPW groups. **(B–I)** The percentages of V1+NKG2D+, V1+NKP30+, V1+NKP46+, V1+PD-1+, V2+NKG2D+, V2+NKP30+ and V2+NKP46+ γδ T cells were not significantly different among the three groups. The percentage of Vdelta2+PD-1+ γδ T cells was significantly different between the RPL and NP groups (P < 0.05). **(I)** The mean ± 1.96 SD was used to measure the reference range for the normally distributed data. The median and 5^th^/95^th^ percentiles represent the lower/upper limits of the reference range for skewed distribution data. Significance levels were set to *P* < 0.05 (*), and *ns* means not significant.
