# Erratum: Systemic Characterization of Novel Immune Cell Phenotypes in Recurrent Pregnancy Loss

**DOI:** 10.3389/fimmu.2021.722805

**Published:** 2021-06-23

**Authors:** Frontiers Production Office

**Affiliations:** Frontiers Media SA, Lausanne, Switzerland

**Keywords:** recurrent pregnancy loss, flow cytometry, peripheral T helper cell, immune cell, NK cell, γδT cell

Due to a production error, additional proof corrections submitted by the author were not implemented in the final version. Corrections have therefore been made throughout the article.

There was an error in **Table 1**, **Table 2**, **Figure 1**, and **Figure 3**. Instead of “Characteristics of 12 immune parameters with significant differences in NPW, NP and RPL groups”, **Table 1** should say “Characteristics of 11 immune parameters with significant differences in NPW, NP and RPL groups”. Therefore, references to “V2^+^PD^-^1^+^” were removed in these tables and figures, as well as throughout the text, including the **Abstract**, **Introduction** and **Results**.

The corrected tables and figures appear below.

Additionally, **Supplementary Figure 1**, **Supplementary Figure 3**, and **Supplementary Table 2** were not the latest versions. The correct versions appear below.

The publisher apologizes for these mistakes. The original version of this article has been updated.

**Table 1:** Characteristics of 11 immune parameters with significant differences in NPW, NP and RPL groups.

	Mean/ Median	SD/ Range	95% upper limit	5% lower limit	NPW			NP			RPL		
					Higher (%)	In (%)	Lower (%)	Higher (%)	In (%)	Lower (%)	Higher (%)	In (%)	Lower (%)
Naïve CD4^+^ T cells^a^	21.12	12.96	46.52	-4.28	4	96	0	4	96	0	8	92	0
Central memory CD4^+^ T cellsb	7.83	5.88- 9.96	16.27	1.97	4	92	4	4	94	2	24	76	0
Terminal different CD4^+^ T cells^b^	18.70	13.35- 25.95	40.87	6.57	4	92	4	4	94	2	0	72	28
Effective memory CD4^+^ T cells^a^	50.74	11.76	73.79	27.69	2	96	2	4	96	0	0	94	6
Naïve CD8^+^ T cells^b^	21.55	13.33- 32.53	49.78	6.61	4	92	4	0	94	6	10	90	0
Immature NK cell^b^	87.35	69.80- 92.23	96.08	24.56	4	92	4	0	98	2	2	58	40
Mature NK cells^b^	12.35	7.07- 29.63	75.45	3.86	4	92	4	2	98	0	40	56	4
Vdelta 1^+a^	43.23	21.18	84.74	1.72	2	98	0	2	98	0	32	68	0
Vdelta 2^+a^	56.69	21.2	98.24	15.14	0	98	2	0	98	2	0	68	32
Vdelta 1^+^/Vdelta 2^+b^	0.74	0.35- 1.56	4.86	0.12	4	94	2	4	94	2	34	64	2
Peripheral T helper cells^b^	60.97	5.88	72.49	49.45	0	98	2	0	100	0	0	96	4

^a^mean/SD; ^b^median/range.

**Table 2:** Potential functions of significantly different immune cell subsets.

Cell subsets	Potential functions	Ref
Naïve CD4^+^ T cells	Naïve CD4^+^ T cells differentiate to effector T cells and subsequently develop into long-lived memory T cells.	(32, 33)
Naïve CD8^+^ T cells	Naïve CD8^+^ T cells differentiate to effector T cells and subsequently develop into long-lived memory T cells.	(32, 33)
Terminal differentiated CD4^+^ T cells	Terminally differentiated CD4^+^ T cells are associated with protection, though they do not have the ability of renewal and differentiation	(34)
Central memory CD4^+^ T cells	Central memory CD4^+^ T cells mediate reactive memory, readily proliferate, and differentiate to effector cells and produce large amounts of IFN-γ or IL-4 in response to antigenic stimulation.	(35, 36)
Effective memory CD4^+^ T cells	Effective memory CD4^+^ T cells mediate protective memory, display immediate effector function.	(35, 36)
Immature NK cells	Immature NK cells have low cytotoxicity and high production of cytokines and chemokines, including M-CSF and GM-CSF.	(37)
Mature NK cells	Mature NK cells are functionally well known for their potent cytotoxic activity	(37–39)
Vδ1^+^ T cells	Vδ1^+^ T cells possess both regulatory and effector properties. Vδ1 T cells could kill tumor cells and have pro-inflammatory properties.	(40–42)
Vδ2^+^ T cells	Vδ2^+^ T cells exert a cytolytic effect against pathogenic properties.	(40)
Peripheral T helper cells (T_PH_)	T_PH_ cells are uniquely antigen-specific T cells with increased expression of genes associated with B cell functions.	(43–45)

**Figure 1:**
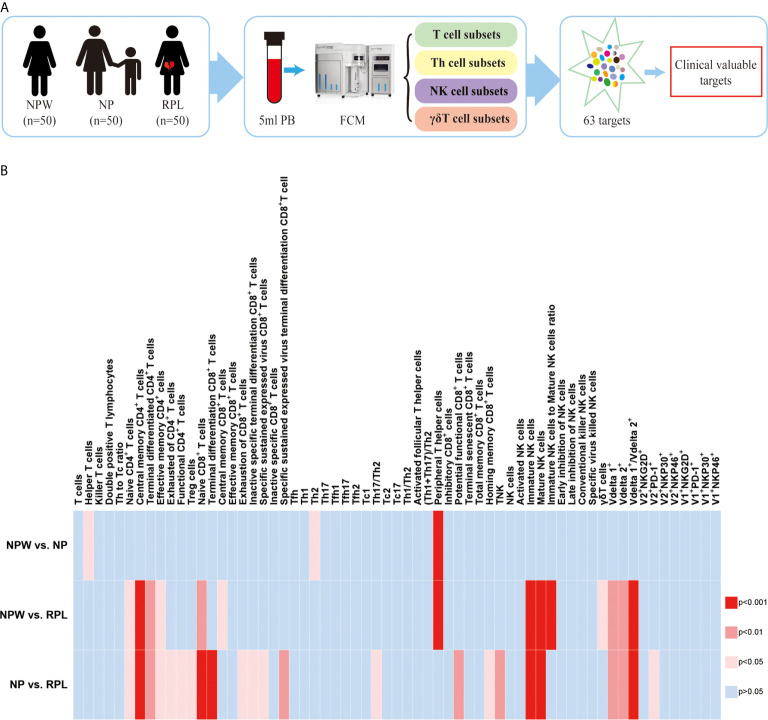
Study design and the overview of the different immunological parameters among the three groups. **(A)** Schedule of the study. Three groups were included in our study: NPW group (women who were never pregnant, n=50), NP group (women with the history of normal pregnancy, n=50) and RPL group (women with the history of RPL, n=50). Peripheral blood mononuclear cells (PBMCs) were isolated from 5 ml peripheral blood. Total 63 immune cell subsets were simultaneously detected by flow cytometry, including T cell, NK cell and gd T cell subsets. By analyzing the data, clinical-relevant immune parameters were finally identified. **(B)** Heat map of the significantly changed immune parameters. The 63 immunological parameters were compared among the three groups and presented as the heat map, which can directly show the differences. The colors represent the different significance among the comparisons. The deeper the color is red, the larger the differences are. The blue represents no significance. NPW, women never pregnant; NP, women with a history of normal pregnancy; RPL, women with ahistory of RPL.

**Figure 3:**
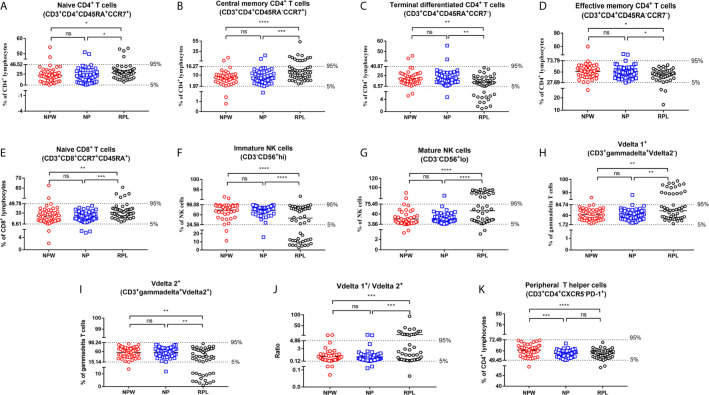
Reference ranges for the significantly changed immune parameters. **(A–J)** Reference ranges for significantly changed immune parameters related to RPL. The percentages of naïve CD4^+^ T cells **(A)**, central memory CD4^+^ T cells **(B)**, naïve CD8^+^ T cells **(E)**, mature NK cells **(G)**, Vδ1^+^ T cells **(H)** and the ratio of Vδ1^+^ T cells/Vδ2^+^ T cells **(J)** were significantly higher in the RPL group than those in the NPW and NP groups. The percentages of terminal differentiated CD4^+^ T cells **(D)**, effective memory CD4^+^ T cells **(D)**, immature NK cells **(F)** and Vδ2^+^ T cells **(I)** were significantly lower in the RPL group than those in the NPW and NP groups. The mean ^+^ 1.96 SD was used to measure the reference ranges for the normally distributed data. Median and 5th/95th percentiles represented the lower/upper limit to set up the reference ranges for skewed distribution data. In 14%- 40% of women with RPL, the percentages of central memory CD4^+^ T cells **(B)**, mature NK cells **(G)**, the ratio of Vδ1^+^ T cells/Vδ2^+^ T cells **(J)**, and Vδ1^+^ T cells **(H)** were above the 95th percentile limit. In 28% - 40% of women with RPL group, the percentages of terminally differentiated CD4^+^ T cells **(C)**, immature NK cells **(F)** and Vδ2^+^ T cells **(I)** were below the 5th percentile limit. The percentages of these different immunological parameters in the NP group were similar to those in the NPW group, and most were within the 5th percentile limit and 95th percentile limit. **(K)** Reference ranges for significantly changed immune parameters related to pregnancies. The percentage of TPH was significantly low in the NP and RPL groups compared with the NPW group. Vdelta1: Vd1; Vdelta2: Vd2. Significance levels were set to *P < 0.05, **P < 0.01, ***P < 0.001, and ****P < 0.0001, and ns means not significant.

## Supplementary Material

Supplementary Figure 1 Reference ranges for nonsignificant immune parameters in CD4+ and CD8+ T cells. **(A)** Th1 and Th17 cells were not significantly different in the RPL vs. NP and RPL vs. NPW groups. Th2 cells were significantly different in the NP vs. NPW groups but not in the RPL vs. NP and RPL vs. NPW groups. Treg cells were significantly different in the RPL vs. NP groups but not in the RPL vs. NPW groups. **(B)** T cells (a), killer T cells (b), effective memory CD8+ T cells (e), inactive specific CD8+ T cells (i), inhibitory CD8+ T cells (k), terminally senescent CD8+ T cells (m) and total memory CD8+ T cells (n) were not significantly different among the three groups. Some of the parameters were lower or higher than the 5th/95th percentile limits. Terminal differentiation CD8+ T cells (c), exhaustion of CD8+ T cells (f), inactive specific terminal differentiation CD8+ T cells (g), specific sustained expressed virus CD8+ T cells (h), specific sustained expressed virus terminal differentiation CD8+ T cells (j), potential functional CD8+ T cells (l), and homing memory CD8+ T cells (o) were significantly different in the RPL vs. NP groups but not in the RPL vs. NPW groups. Central memory CD8+ T cells (d) were significantly different in the NPW vs. RPL groups but not in RPL vs. NP groups. **(C)** CD4+ T cell DP T lymphocytes and activated Tfh cells were not significantly different among the three groups. Helper T cells were significantly different in the NP vs. NPW groups. The exhaustion of CD4+ T cells and functional CD4+ T cells was significant in the RPL vs. NP groups but not in the RPL vs. NPW groups. **(D)** Tfh cells (a) and cytotoxic T (Tc) cells (Tfh, Tfh1, Tfh2, Tfh17, Tc1, Tc2 and Tc17) (b–g) were not significantly different among the three groups. **(E)** The Th to Tc ratios (a), Th1/Th2 ratios (c) and (Th1+Th17)/Th2 ratios (d) were nonsense parameters among the three groups. Th17/Th2 (b) was significantly different in the RPL vs. NP groups. The ratio of immature NK cells to mature NK cells (e) was significantly different in the RPL vs. NPW groups. The mean ± 1.96 SD was used to measure the reference range for the normally distributed data. The median and 5th/95th percentiles represent the lower/upper limits of each reference range for skewed distribution data. Significance levels were set to *P < 0.05, **P < 0.01, and ***P < 0.001 and ns means not significant.

Supplementary Figure 3 Reference ranges for nonsignificant immune parameters in gd T cells. **(A)** γδ T cells were significantly different in the RPL vs. NPW groups. **(B–I)** The percentages of V1+NKG2D+, V1+NKP30+, V1+NKP46+, V1+PD-1+, V2+NKG2D+, V2+NKP30+ and V2+NKP46+ γδ T cells were not significantly different among the three groups. The percentage of Vdelta2+PD-1+ γδ T cells was significantly different between the RPL and NP groups (P < 0.05). **(I)** The mean ± 1.96 SD was used to measure the reference range for the normally distributed data. The median and 5th/95th percentiles represent the lower/upper limits of the reference range for skewed distribution data. Significance levels were set to P < 0.05 (*), and *ns* means not significant.
