# Supplementary figures and images for: Erratum: Systemic Characterization of Novel Immune Cell Phenotypes in Recurrent Pregnancy Loss
Source: Front Immunol. 2021 Jun 23;12:722805. doi: 10.3389/fimmu.2021.722805 (PMC8262454; doi:10.3389/fimmu.2021.722805)

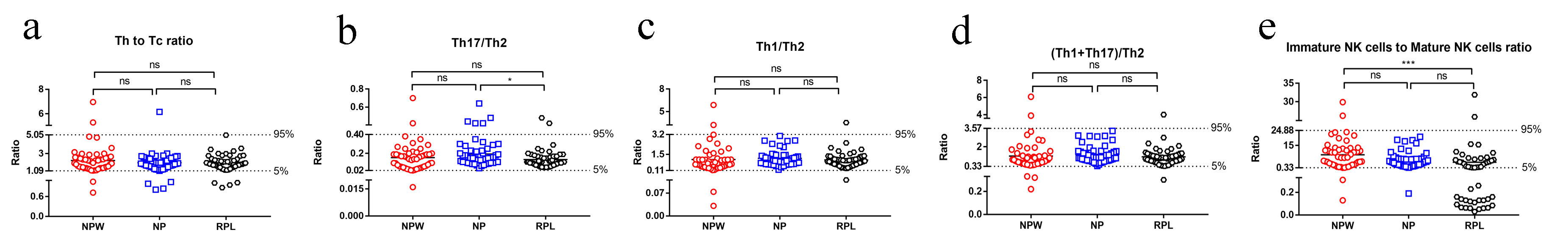

Supplement: Supplementary Figure 1 — Reference ranges for nonsignificant immune parameters in CD4+ and CD8+ T cells. (A) Th1 and Th17 cells were not significantly different in the RPL vs. NP and RPL vs. NPW groups. Th2 cells were significantly different in the NP vs. NPW groups but not in the RPL vs. NP and RPL vs. NPW groups. Treg cells were significantly different in the RPL vs. NP groups but not in the RPL vs. NPW groups. (B) T cells (a), killer T cells (b), effective memory CD8+ T cells (e), inactive specific CD8+ T cells (i), inhibitory CD8+ T cells (k), terminally senescent CD8+ T cells (m) and total memory CD8+ T cells (n) were not significantly different among the three groups. Some of the parameters were lower or higher than the 5th/95th percentile limits. Terminal differentiation CD8+ T cells (c), exhaustion of CD8+ T cells (f), inactive specific terminal differentiation CD8+ T cells (g), specific sustained expressed virus CD8+ T cells (h), specific sustained expressed virus terminal differentiation CD8+ T cells (j), potential functional CD8+ T cells (l), and homing memory CD8+ T cells (o) were significantly different in the RPL vs. NP groups but not in the RPL vs. NPW groups. Central memory CD8+ T cells (d) were significantly different in the NPW vs. RPL groups but not in RPL vs. NP groups. (C) CD4+ T cell DP T lymphocytes and activated Tfh cells were not significantly different among the three groups. Helper T cells were significantly different in the NP vs. NPW groups. The exhaustion of CD4+ T cells and functional CD4+ T cells was significant in the RPL vs. NP groups but not in the RPL vs. NPW groups. (D) Tfh cells (a) and cytotoxic T (Tc) cells (Tfh, Tfh1, Tfh2, Tfh17, Tc1, Tc2 and Tc17) (b–g) were not significantly different among the three groups. (E) The Th to Tc ratios (a), Th1/Th2 ratios (c) and (Th1+Th17)/Th2 ratios (d) were nonsense parameters among the three groups. Th17/Th2 (b) was significantly different in the RPL vs. NP groups. The ratio of immature NK cells to mature NK cells [file Image_1.tif]

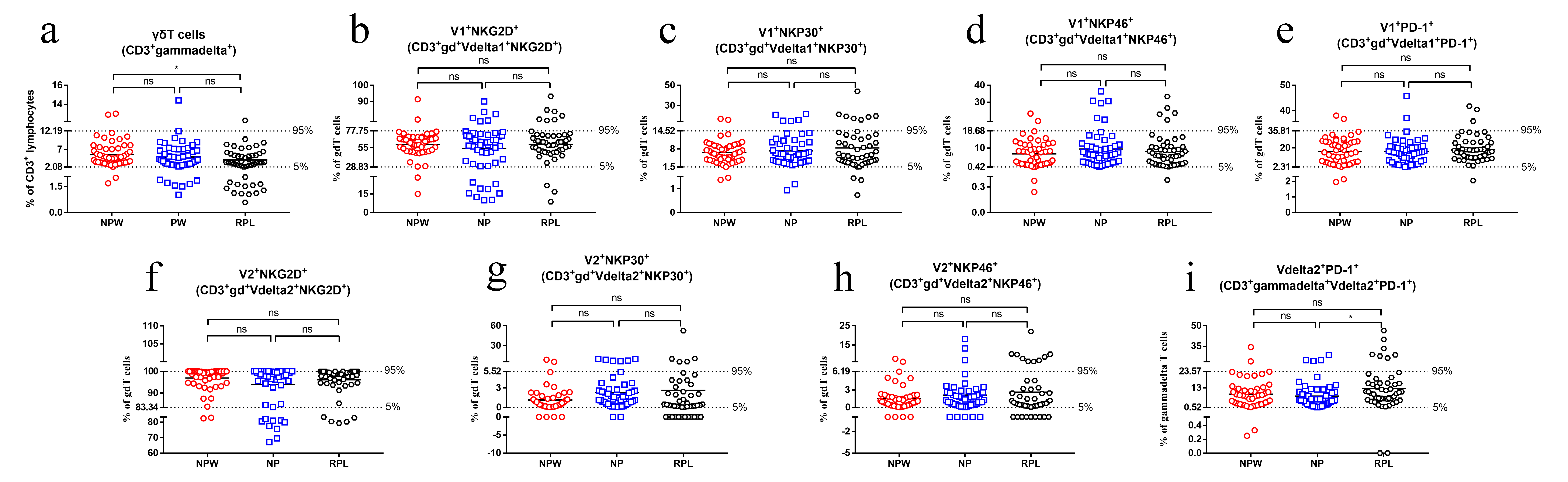

Supplement: Supplementary Figure 3 — Reference ranges for nonsignificant immune parameters in gd T cells. (A) γδ T cells were significantly different in the RPL vs. NPW groups. (B–I) The percentages of V1+NKG2D+, V1+NKP30+, V1+NKP46+, V1+PD-1+, V2+NKG2D+, V2+NKP30+ and V2+NKP46+ γδ T cells were not significantly different among the three groups. The percentage of Vdelta2+PD-1+ γδ T cells was significantly different between the RPL and NP groups (P < 0.05). (I) The mean ± 1.96 SD was used to measure the reference range for the normally distributed data. The median and 5th/95th percentiles represent the lower/upper limits of the reference range for skewed distribution data. Significance levels were set to P < 0.05 (*), and ns means not significant. [file Image_3.tif]
