# Supplementary material for: Erratum: Systemic Characterization of Novel Immune Cell Phenotypes in Recurrent Pregnancy Loss
Source: Front Immunol. 2021 Jun 23;12:722805. doi: 10.3389/fimmu.2021.722805 (PMC8262454; doi:10.3389/fimmu.2021.722805)
Supplement: Supplementary file 3 [file Table_2.docx]

Table S2 Characteristics of 51 immunological parameters in NPW, NP and RPL groups

|  | Mean/ Median | SD/ Range | 95% upper limit | 5% lower limit | NPW | | | NP | | | RPL | | |
| --- | --- | --- | --- | --- | --- | --- | --- | --- | --- | --- | --- | --- | --- |
|  |  |  |  |  | Higher (%) | In (%) | Lower (%) | Higher (%) | In (%) | Lower (%) | Higher (%) | In (%) | Lower (%) |
| T cells ^b^ | 72.65 | 65.45-78.85 | 82.47 | 50.38 | 4 | 92 | 4 | 12 | 88 | 0 | 6 | 94 | 0 |
| Helper T cells ^a^ | 59.02 | 11.54 | 81.64 | 36.40 | 2 | 94 | 4 | 2 | 98 | 0 | 0 | 100 | 0 |
| Killer T cells ^a^ | 30.59 | 10.10 | 50.39 | 10.79 | 2 | 96 | 0 | 0 | 100 | 0 | 0 | 100 | 0 |
| Double positive T lymphocytes ^b^ | 1.85 | 1.15-2.98 | 6.25 | 0.48 | 4 | 92 | 4 | 8 | 92 | 0 | 2 | 92 | 6 |
| Th to Tc ratio ^b^ | 1.95 | 1.40-2.56 | 5.05 | 1.09 | 4 | 92 | 4 | 2 | 90 | 8 | 0 | 92 | 8 |
| Exhausted of CD4^+^ T cell ^b^ | 2.34 | 1.50-4.23 | 12.66 | 0.73 | 4 | 94 | 2 | 6 | 92 | 2 | 2 | 74 | 24 |
| Functional CD4^+^ T cells ^b^ | 97.65 | 95.78-98.48 | 99.30 | 87.33 | 0 | 96 | 4 | 2 | 92 | 6 | 22 | 76 | 2 |
| Treg cells ^b^ | 3.52 | 2.87-5.03 | 10.26 | 1.72 | 4 | 92 | 4 | 10 | 82 | 8 | 0 | 88 | 12 |
| Terminal differentiated CD8^+^ T cells ^b^ | 35.25 | 26.00-54.85 | 66.69 | 17.61 | 4 | 92 | 4 | 6 | 94 | 0 | 2 | 82 | 16 |
| Central memory CD8^+^ T cells ^b^ | 0.60 | 0.38-0.90 | 2.70 | 0.15 | 4 | 92 | 4 | 6 | 94 | 0 | 8 | 92 | 0 |
| Effective memory CD8^+^ T cells ^a^ | 36.20 | 13.88 | 63.40 | 8.99 | 0 | 100 | 0 | 2 | 98 | 0 | 0 | 100 | 0 |
| Exhausted of CD8^+^ T cells ^b^ | 11.75 | 6.04-41.00 | 52.52 | 4.59 | 4 | 92 | 4 | 12 | 86 | 2 | 6 | 74 | 20 |
| Inactive specific terminal differentiation CD8^+^ T cells ^a^ | 53.51 | 16.86 | 86.56 | 20.46 | 4 | 94 | 2 | 10 | 86 | 4 | 20 | 72 | 8 |
| Specific sustained expressed virus CD8^+^ T cells ^b^ | 27.40 | 19.60-35.75 | 56.33 | 8.17 | 4 | 92 | 4 | 12 | 72 | 16 | 6 | 74 | 20 |
| Inactive specific CD8^+^ T cells ^b^ | 72.60 | 64.25-80.40 | 91.85 | 43.67 | 4 | 92 | 4 | 16 | 72 | 12 | 20 | 72 | 8 |
| Specific sustained expressed virus terminal differentiation CD8^+^ T cells ^a^ | 46.49 | 16.86 | 79.54 | 13.44 | 2 | 94 | 4 | 4 | 86 | 10 | 8 | 72 | 20 |
| Tfh ^a^ | 19.52 | 4.14 | 27.63 | 11.41 | 2 | 96 | 2 | 4 | 96 | 0 | 10 | 90 | 0 |
| Th1 ^b^ | 12.65 | 8.80-19.80 | 31.15 | 3.26 | 4 | 92 | 4 | 0 | 100 | 0 | 2 | 96 | 2 |
| Th2 ^b^ | 16.95 | 14.55-21.33 | 31.70 | 9.04 | 2 | 94 | 4 | 2 | 90 | 8 | 2 | 92 | 6 |
| Th17 ^b^ | 1.99 | 0.94-4.19 | 7.67 | 0.41 | 4 | 92 | 4 | 2 | 98 | 0 | 0 | 100 | 0 |
| Tfh1 ^a^ | 13.05 | 5.41 | 23.65 | 2.45 | 2 | 96 | 2 | 4 | 96 | 0 | 6 | 94 | 0 |
| Tfh17 ^b^ | 5.35 | 3.85-7.55 | 11.68 | 2.32 | 4 | 92 | 4 | 4 | 96 | 0 | 2 | 98 | 0 |
| Tfh2 ^a^ | 37.19 | 8.14 | 53.14 | 21.23 | 2 | 96 | 2 | 0 | 96 | 4 | 2 | 98 | 0 |
| Tc1 ^a^ | 31.77 | 14.79 | 60.76 | 2.78 | 0 | 98 | 2 | 0 | 100 | 0 | 0 | 100 | 0 |
| Th17/Th2 ^b^ | 0.14 | 0.06-0.20 | 0.40 | 0.02 | 4 | 94 | 2 | 10 | 90 | 0 | 4 | 96 | 0 |
| Tc2 ^b^ | 5.86 | 3.76-7.90 | 12.33 | 1.41 | 4 | 92 | 4 | 0 | 94 | 6 | 6 | 94 | 0 |
| Tc17 ^b^ | 8.27 | 3.83-13.13 | 20.84 | 0.94 | 4 | 92 | 4 | 6 | 84 | 10 | 2 | 96 | 2 |
| Th1/Th2 ^b^ | 0.73 | 0.42-1.07 | 3.20 | 0.11 | 4 | 92 | 4 | 0 | 100 | 0 | 2 | 96 | 2 |
| Activated follicular T helper ^a^ | 15.62 | 3.35 | 22.19 | 9.05 | 4 | 96 | 0 | 4 | 94 | 2 | 8 | 90 | 2 |
| (Th1+Th17)/Th2 ^b^ | 0.89 | 0.56-1.30 | 3.57 | 0.33 | 4 | 92 | 6 | 0 | 100 | 0 | 2 | 96 | 2 |
| Inhibitory CD8^+^ T cells ^b^ | 15.60 | 12.95-22.00 | 33.54 | 8.82 | 4 | 92 | 4 | 6 | 92 | 2 | 4 | 92 | 4 |
| Potential functional CD8^+^ T cells ^a^ | 69.22 | 12.03 | 92.80 | 45.64 | 2 | 96 | 2 | 0 | 96 | 4 | 0 | 100 | 0 |
| Terminally senescent CD8^+^ T cells ^b^ | 15.40 | 8.97-24.03 | 35.81 | 3.24 | 4 | 92 | 4 | 8 | 92 | 0 | 10 | 90 | 0 |
| Total memory CD8^+^ T cells ^b^ | 2.75 | 1.98-4.07 | 8.35 | 0.56 | 4 | 92 | 4 | 2 | 96 | 2 | 6 | 90 | 4 |
| Homing memory CD8^+^ T cells ^a^ | 58.12 | 17.05 | 91.54 | 24.70 | 2 | 96 | 2 | 2 | 96 | 2 | 4 | 96 | 0 |
| TNK ^b^ | 5.64 | 3.83-9.25 | 14.25 | 1.43 | 4 | 92 | 4 | 4 | 94 | 2 | 2 | 98 | 0 |
| NK cells ^b^ | 9.08 | 6.57-14.05 | 21.06 | 3.24 | 4 | 92 | 4 | 2 | 96 | 2 | 6 | 94 | 0 |
| Activated NK cell ^b^ | 69.25 | 60.50-82.35 | 90.56 | 34.98 | 4 | 92 | 4 | 2 | 86 | 12 | 12 | 78 | 10 |
| Immature NK cells to Mature NK cells ratio ^b^ | 7.05 | 2.36-13.00 | 24.88 | 0.33 | 4 | 92 | 8 | 0 | 98 | 2 | 4 | 56 | 40 |
| Early inhibition of NK cells ^a^ | 49.31 | 17.31 | 83.24 | 15.38 | 2 | 98 | 0 | 0 | 98 | 2 | 0 | 100 | 0 |
| Late inhibition of NK cells ^b^ | 6.19 | 3.59-11.48 | 28.76 | 0.005 | 4 | 92 | 4 | 4 | 96 | 0 | 0 | 98 | 2 |
| Conventional killer NK cells ^a^ | 67.87 | 17.66 | 102.48 | 33.26 | 0 | 96 | 4 | 0 | 96 | 4 | 0 | 92 | 8 |
| Specific virus killed NK cells ^b^ | 81.75 | 61.95-90.03 | 95.25 | 36.51 | 4 | 92 | 4 | 0 | 98 | 2 | 2 | 92 | 6 |
| γδT ^b^ | 4.68 | 3.29-7.40 | 12.19 | 2.08 | 4 | 92 | 4 | 2 | 84 | 14 | 2 | 72 | 26 |
| Vδ2^+^ NKG2D^+ b^ | 99.60 | 94.78-100 | 100 | 83.34 | 0 | 96 | 4 | 0 | 82 | 18 | 0 | 90 | 10 |
| Vδ2^+^ PD-1^+ b^ | 7.91 | 2.62-12.50 | 23.57 | 0.52 | 4 | 92 | 4 | 8 | 92 | 0 | 14 | 86 | 0 |
| Vδ2^+^ NKP30^+ b^ | 0.49 | 0.16-1.36 | 5.52 | 0 | 4 | 96 | 0 | 12 | 88 | 0 | 10 | 90 | 0 |
| Vδ2^+^ NKP46^+ b^ | 0.88 | 0.30-1.87 | 6.19 | 0 | 4 | 96 | 0 | 6 | 94 | 0 | 18 | 82 | 0 |
| Vδ1^+^ NKG2D^+ b^ | 61.05 | 50.43-68.33 | 77.75 | 28.83 | 4 | 92 | 4 | 12 | 70 | 18 | 14 | 80 | 6 |
| Vδ1+ PD-1^+ b^ | 16.05 | 6.78-25.95 | 35.81 | 2.31 | 4 | 92 | 4 | 4 | 96 | 0 | 6 | 92 | 2 |
| Vδ1^+^ NKP30^+ b^ | 6.49 | 4.13-9.14 | 14.52 | 1.50 | 4 | 92 | 4 | 10 | 86 | 4 | 14 | 80 | 6 |
| Vδ1^+^ NKP46^+ b^ | 6.16 | 1.99-11.63 | 18.68 | 0.42 | 4 | 92 | 4 | 12 | 88 | 0 | 10 | 88 | 2 |

a: mean/SD；b:median/range
